# Supplementary material for: Some poleward movement of British native vascular plants is occurring, but the fingerprint of climate change is not evident
Source: PeerJ. 2013 May 28;1:e77. doi: 10.7717/peerj.77 (PMC3669268; doi:10.7717/peerj.77)
Supplement: Table S1 — The direction and distance moved by the centre of mass and the mean occupancy probability change of native species in southern England, northern England, Wales and Scotland. Occupancy probabilities are calculated for 4 km2 grid squares of the Ordnance Survey of the United Kingdom. [file peerj-01-77-s001.pdf]

	southern England			northern England			Wales			Scotland		
Taxon	Direction (degrees)	Distance (km)	Mean Occupancy Change	Direction (degrees)	Distance (km)	Mean Occupancy Change	Direction (degrees)	Distance (km)	Mean Occupancy Change	Direction (degrees)	Distance (km)	Mean Occupancy Change
Acer campestre	74	2.1	0.071	322	36.1	0.123	288	30.7	-0.058	143	11.6	0.003
Achillea millefolium	119	0.2	-0.006	27	0.9	-0.021	8	1.6	-0.019	116	1.5	-0.037
Achillea ptarmica	226	22.5	-0.041	333	3.9	-0.120	50	2.4	-0.008	353	5.3	-0.033
Adiantum capillus-veneris	83	5.3	-0.001									
Adoxa moschatellina	137	6.6	-0.008	314	10.7	-0.023	255	4.4	-0.101	107	18.6	-0.038
Aethusa cynapium	56	13.9	0.019	262	1.1	0.046	196	2.2	-0.011	19	9.6	-0.005
Agrimonia eupatoria	103	6.5	-0.027	279	20.6	0.010	163	0.9	-0.131	143	9.7	-0.009
Agrimonia procera	115	23.2	-0.003				255	1.3	0.026	277	3.6	0.003
Agrostis capillaris	308	1.7	0.055	298	4.6	-0.167	69	0.5	0.059	319	0.5	-0.013
Agrostis curtisii	231	65.9	-0.010									
Agrostis stolonifera	254	0.6	0.037	136	0.6	0.004	200	4.4	0.004	2	3.0	-0.017
Aira caryophyllea	241	28.2	-0.018	16	6.3	-0.021	181	13.4	0.042	343	4.9	-0.028
Aira praecox	116	12.3	0.013	138	19.8	-0.037	355	9.0	0.149	351	4.2	0.001
Ajuga pyramidalis										334	18.7	-0.008
Ajuga reptans	159	1.9	-0.012	328	20.0	-0.070	343	11.6	-0.156	184	5.4	-0.019
Alchemilla alpina										175	26.2	-0.085
Alchemilla filicaulis	246	11.2	-0.023	194	1.3	-0.061	185	46.2	-0.072	112	4.6	-0.016
Alisma lanceolatum	31	6.0	0.006									
Alisma plantago-aquatica	167	3.5	-0.036	165	8.8	0.081	198	10.5	0.041	17	13.0	-0.008
Alliaria petiolata	53	0.7	0.036	38	2.7	0.057	54	10.1	-0.029	288	9.2	0.030
Allium oleraceum	306	12.6	0.002	345	2.4	0.003						
Allium schoenoprasum	30	40.1	0.008									
Allium scorodoprasum				97	1.8	0.010						
Allium ursinum	139	5.2	0.068	328	15.4	-0.039	126	2.1	-0.072	184	10.9	-0.023
Allium vineale	212	8.5	0.058	20	4.1	0.037	346	14.5	-0.026	135	2.8	-0.003
Alnus glutinosa	144	0.1	0.072	357	0.6	-0.018	96	3.9	-0.127	171	7.5	0.009
Alopecurus aequalis	217	16.5	0.001									
Alopecurus bulbosus	144	14.5	0.001									
Alopecurus geniculatus	226	3.8	-0.043	282	1.1	-0.102	346	2.9	-0.014	333	2.4	-0.047
Alopecurus pratensis	58	8.5	-0.039	302	0.8	-0.067	10	2.6	-0.194	344	14.5	-0.055
Althaea officinalis	120	53.5	0.003									
Anacamptis morio	159	6.5	-0.012	291	2.4	-0.007	51	1.4	0.020			
Anacamptis pyramidalis	207	5.4	0.039	76	13.7	0.028	196	4.3	0.060			
Anagallis arvensis	111	7.2	0.021	303	6.4	0.086	301	2.9	0.135	166	4.8	0.004
Anagallis tenella	256	31.1	-0.020	212	2.7	-0.040	266	9.8	0.002	273	23.5	-0.004
Andromeda polifolia				304	1.9	0.002						
Anemone nemorosa	211	7.7	-0.002	321	17.2	-0.131	71	9.9	-0.052	211	2.5	-0.046
Angelica sylvestris	35	3.3	-0.026	309	5.1	-0.098	333	1.6	-0.057	299	1.0	-0.001
Antennaria dioica										326	14.4	-0.009
Anthoxanthum odoratum	74	0.8	-0.027	320	2.9	-0.107	303	0.2	-0.022	330	1.4	-0.034
Anthriscus caucalis	175	13.5	0.038									
Anthriscus sylvestris	48	1.8	-0.013	342	3.6	0.042	17	7.1	-0.064	334	33.9	0.051
Anthyllis vulneraria	243	5.5	-0.034	348	17.9	-0.058	247	18.1	0.042	356	8.0	0.044
Apium graveolens	250	17.5	-0.001	26	3.3	0.006						
Apium inundatum	258	1.7	0.007	141	0.8	-0.001	153	1.4	0.013	132	11.0	-0.017
Apium nodiflorum	307	2.7	0.015	163	19.7	0.146	301	1.6	0.122	205	7.9	-0.017

Aquilegia vulgaris	158	0.7	0.123	163	5.6	0.091	6	1.4	-0.062	12	8.9	0.006
Arabidopsis petraea										295	28.9	0.001
Arabidopsis thaliana	167	3.4	0.064	266	3.8	0.187	274	5.0	0.071	10	16.1	0.024
Arabis hirsuta	18	11.6	-0.015	336	2.5	-0.014	174	3.9	0.007	231	0.4	-0.010
Arctostaphylos alpinus										43	3.2	-0.008
Arctostaphylos uva-ursi										349	16.6	-0.007
Arenaria serpyllifolia	23	0.4	0.032	26	2.0	-0.018	146	10.5	0.013	28	3.8	-0.004
Armeria maritima	56	53.0	0.019	35	5.6	0.021	347	7.4	0.056	328	17.3	0.025
Arrhenatherum elatius	23	0.5	0.020	345	1.4	0.024	312	0.2	0.025	346	8.0	0.046
Arum italicum	76	6.9	0.004	11	2.5	0.014	334	1.2	0.051			
Arum maculatum	120	1.4	0.016	337	8.9	-0.012	213	6.4	-0.058	85	12.1	0.000
Asperula cynanchica	224	12.6	-0.015									
Asplenium ceterach	246	43.8	0.010	33	2.1	0.007	152	3.4	-0.040			
Asplenium obovatum	239	132.5	-0.005									
Asplenium ruta-muraria	242	15.9	0.047	319	4.4	0.142	194	1.0	-0.075	4	1.1	0.044
Asplenium scolopendrium	162	2.6	0.129	306	3.2	0.189	208	2.2	-0.063	185	4.9	-0.019
Asplenium trichomanes	66	28.9	-0.123	344	6.2	0.060	6	0.7	-0.025	246	5.4	0.007
Asplenium viride										148	19.4	-0.017
Astragalus danicus	52	61.2	-0.006	6	2.6	0.011				41	8.3	0.010
Astragalus glycyphyllos	56	14.8	-0.002									
Athyrium distentifolium										122	1.5	-0.008
Athyrium filix-femina	250	30.3	0.022	319	29.4	-0.090	48	0.1	0.015	220	4.0	-0.047
Atriplex littoralis	342	30.2	0.066	235	0.4	0.064				105	20.9	0.012
Atriplex patula	69	13.5	0.005	169	4.7	0.015	228	6.4	-0.044	330	22.9	0.032
Atriplex prostrata	59	4.8	0.150	144	2.2	0.229	44	4.7	0.126	24	15.8	0.051
Atropa belladonna	63	16.6	0.004									
Avenula pratensis	261	5.3	-0.022	342	41.1	0.024				125	16.6	0.000
Avenula pubescens	249	0.3	-0.046	344	11.9	0.008	251	18.6	0.011	298	4.6	-0.003
Baldellia ranunculoides	38	12.6	0.006				196	71.6	0.030	206	4.6	-0.009
Barbarea vulgaris	184	6.3	-0.010	348	1.2	0.039	271	12.4	-0.134	72	10.2	-0.019
Bellis perennis	54	0.9	0.007	311	0.7	-0.002	356	0.8	-0.031	38	1.1	0.023
Berberis vulgaris	53	7.9	-0.003	315	5.4	-0.009				107	8.8	-0.005
Berula erecta	53	19.0	0.017	267	2.0	0.017	0	31.2	0.009	28	18.7	-0.003
Beta vulgaris	35	46.9	0.057	131	13.3	0.022	262	10.7	0.102	200	5.9	0.002
Betonica officinalis	234	34.9	-0.096	291	5.9	-0.085	170	5.0	-0.121			
Betula nana										15	1.5	-0.007
Betula pendula	174	2.1	0.068	279	4.6	0.033	48	11.0	-0.039	68	9.6	-0.027
Betula pubescens	318	7.1	0.038	302	4.0	-0.051	98	1.5	-0.015	231	1.3	-0.009
Bidens cernua	228	6.4	-0.012				236	22.3	-0.004			
Bidens tripartita	194	4.3	-0.016	359	1.3	0.014	178	13.0	-0.031			
Blackstonia perfoliata	149	8.2	0.013	332	12.6	0.056	170	6.9	0.033			
Blechnum spicant	236	71.0	-0.006	318	28.4	-0.078	63	5.7	-0.044	267	4.7	0.036
Blysmus compressus	32	7.8	0.000	347	3.6	0.000						
Botrychium lunaria	254	9.8	-0.001	3	5.2	-0.024				14	3.3	0.029
Brachypodium sylvaticum	65	3.7	0.072	319	12.9	0.110	279	3.6	-0.079	242	3.4	0.022
Brassica nigra	149	5.2	0.032	155	16.5	0.022	270	3.0	0.034			
Brassica oleracea	71	13.4	0.004									
Briza media	89	3.1	-0.037	320	7.5	-0.109	118	2.9	-0.071	133	37.2	-0.032
Bromopsis erecta	233	8.3	-0.031	111	32.7	-0.004						
Bromopsis ramosa	174	5.0	-0.039	314	9.5	-0.033	247	9.4	-0.167	26	18.0	-0.011

Bromus commutatus	272	11.1	0.051									
Bromus hordeaceus	65	7.5	0.067	150	6.5	0.135	354	1.9	-0.054	57	3.7	-0.020
Bromus racemosus	103	13.5	0.027									
Bryonia dioica	57	26.6	0.020	144	43.9	0.090						
Bupleurum tenuissimum	158	54.3	0.003									
Butomus umbellatus	252	10.9	-0.014	22	1.1	0.030						
Buxus sempervirens	60	8.7	0.001	109	7.1	0.037	25	4.6	-0.010	120	20.3	0.006
Calamagrostis canescens	189	26.1	-0.001	67	1.9	0.041						
Calamagrostis epigejos	52	36.6	0.047	122	4.2	0.027						
Calluna vulgaris	240	24.6	-0.039	311	23.2	-0.165	348	2.9	0.005	332	3.8	0.018
Caltha palustris	60	2.3	-0.016	324	8.0	-0.131	354	2.5	-0.087	155	1.0	-0.070
Calystegia sepium	62	3.9	0.031	174	4.6	0.277	285	1.0	-0.027	6	11.3	0.057
Campanula glomerata	343	16.0	-0.037	305	3.0	0.003						
Campanula latifolia	30	38.0	-0.009	343	17.4	-0.051				20	13.2	-0.023
Campanula patula	304	34.7	0.000									
Campanula trachelium	100	7.5	-0.022									
Cardamine amara	36	26.7	0.007	311	23.8	0.043				145	6.9	-0.020
Cardamine bulbifera	139	43.2	0.003									
Cardamine flexuosa	78	5.5	0.072	333	0.2	-0.041	329	1.0	-0.125	325	1.3	-0.019
Cardamine hirsuta	96	3.9	0.107	81	2.9	0.011	66	0.8	-0.018	90	6.4	-0.079
Cardamine impatiens	177	4.8	0.002									
Cardamine pratensis	221	4.3	0.019	305	6.7	-0.131	266	2.4	-0.093	2	0.9	-0.022
Carduus crispus	66	15.9	-0.058	135	8.6	0.092	94	2.2	-0.076	63	6.9	-0.011
Carduus nutans	130	3.7	-0.046	300	15.7	-0.009	204	62.6	0.016			
Carduus tenuiflorus	36	39.1	0.029				281	9.7	0.002	348	58.9	0.006
Carex acuta	80	3.4	-0.014	339	4.6	0.010						
Carex acutiformis	356	4.2	-0.008	315	2.7	0.057	2	12.7	-0.027	168	7.5	-0.006
Carex aquatilis										154	19.5	-0.004
Carex atrata										205	46.6	0.001
Carex bigelowii										197	15.5	0.000
Carex binervis	46	20.9	0.022	312	8.8	-0.073	307	5.5	0.094	163	3.4	0.046
Carex canescens	111	4.3	-0.006	129	37.6	-0.005	324	10.5	0.006	256	3.2	-0.063
Carex capillaris										179	34.0	-0.010
Carex caryophyllea	236	27.5	0.020	145	10.6	-0.066	345	5.2	0.033	153	9.0	-0.060
Carex diandra										112	13.4	0.003
Carex dioica				307	32.4	-0.005	337	28.4	0.017	330	12.4	0.036
Carex disticha	279	11.1	0.018	330	2.9	0.012				147	23.1	-0.015
Carex divisa	293	39.6	-0.003									
Carex divulsa	190	9.4	0.212									
Carex echinata	245	80.5	-0.023	261	2.9	-0.082	302	2.3	0.000	225	2.6	0.029
Carex elata	55	78.1	0.010	44	21.5	0.009						
Carex flacca	221	1.9	0.055	326	3.8	-0.008	253	0.9	0.218	6	2.6	0.003
Carex hirta	194	1.9	0.064	166	2.4	0.151	333	7.1	0.082	353	16.1	-0.008
Carex hostiana	241	24.7	0.010	161	5.1	-0.048	248	14.3	0.019	304	7.5	0.016
Carex humilis	11	34.4	-0.005									
Carex laevigata	238	24.8	0.002	324	26.3	-0.026	213	2.2	-0.035	198	6.4	-0.004
Carex lasiocarpa										162	8.2	-0.018
Carex leporina	46	3.2	0.026	302	19.9	-0.086	1	4.3	-0.012	227	0.8	-0.019
Carex limosa										239	1.3	-0.003
Carex magellanica										14	59.1	-0.009

Carex montana	232	25.8	0.003									
Carex muricata	210	14.2	0.043				107	19.8	0.093	358	65.4	0.003
Carex nigra	354	11.0	-0.037	58	1.0	-0.195	69	3.3	-0.052	170	4.6	-0.058
Carex otrubae	232	11.3	0.006	145	19.4	0.133	196	12.0	0.122	239	8.6	0.010
Carex pallescens	217	15.0	-0.004	317	28.8	-0.021	107	0.8	-0.094	229	5.0	-0.024
Carex panicea	290	20.2	-0.021	319	1.8	-0.136	74	7.5	0.086	181	4.5	-0.037
Carex paniculata	242	11.1	0.028	297	4.9	-0.024	93	18.7	-0.031	96	12.5	-0.003
Carex pauciflora										336	11.4	-0.004
Carex pendula	51	5.3	0.261	246	9.5	0.159	193	5.5	0.143	146	7.7	-0.003
Carex pilulifera	253	16.9	0.054	178	12.0	-0.031	337	5.2	0.078	221	1.6	0.010
Carex pseudocyperus	199	6.1	0.038									
Carex pulicaris	251	38.8	-0.002	154	18.7	-0.049	275	10.8	0.018	200	2.8	-0.003
Carex remota	199	1.7	0.086	267	15.2	-0.015	115	7.5	0.067	175	3.4	-0.008
Carex riparia	263	13.7	0.002	140	20.9	0.083	89	9.2	0.036			
Carex rostrata	48	20.4	-0.002	320	17.5	-0.046	81	0.8	0.036	168	0.5	-0.022
Carex saxatilis										121	0.5	-0.003
Carex spicata	73	2.9	0.026	131	5.0	0.044						
Carex strigosa	215	12.6	0.007									
Carex sylvatica	233	14.8	0.063	299	8.6	0.049	164	4.5	-0.046	168	9.9	-0.028
Carex vaginata										196	24.4	-0.024
Carex vesicaria	357	16.4	0.001	131	4.1	-0.009				63	14.0	0.018
Carlina vulgaris	229	19.7	-0.020	23	15.2	-0.027	172	12.2	0.019	265	7.1	-0.004
Carpinus betulus	246	7.1	0.102	298	2.9	0.069	204	46.1	0.024	137	16.0	-0.014
Carum verticillatum							213	38.5	-0.044	152	9.0	-0.026
Catabrosa aquatica	62	70.6	0.003	288	5.3	-0.006				208	3.4	0.016
Catapodium rigidum	230	2.5	0.072	288	37.3	0.049	194	2.7	0.041			
Centaurea nigra	66	0.8	-0.022	18	0.9	0.004	357	0.4	-0.017	183	2.6	-0.002
Centaurea scabiosa	82	11.0	-0.085	71	18.1	-0.021	182	4.7	-0.003			
Centaurium erythraea	91	9.0	0.027	303	5.2	0.022	223	8.3	0.076	217	4.7	0.009
Centunculus minimus	181	40.9	-0.006							221	5.6	0.020
Cephalanthera damasonium	186	16.7	0.001									
Cerastium alpinum										124	11.2	-0.007
Cerastium arvense	56	60.2	-0.010	128	11.8	-0.002				333	11.2	0.001
Cerastium diffusum	45	25.0	0.032	51	3.3	0.005	315	14.3	0.045	7	6.3	0.026
Cerastium fontanum	52	0.6	0.018	321	0.6	-0.023	87	0.2	-0.006	9	1.3	0.031
Cerastium glomeratum	78	7.8	0.146	237	2.2	0.154	2	1.5	0.129	4	7.2	0.062
Cerastium pumilum	241	8.0	0.011									
Cerastium semidecandrum	69	25.1	0.049	124	3.2	0.028	7	4.6	0.031	6	23.8	0.009
Ceratocapnos claviculata	82	41.3	-0.001	349	3.7	0.028	11	8.7	0.046	178	14.5	0.019
Ceratophyllum demersum	245	38.5	0.021	150	5.6	0.039	236	12.6	-0.006			
Ceratophyllum submersum	251	26.8	-0.004									
Chaerophyllum temulum	313	4.5	0.002	277	5.8	0.026	209	6.9	-0.034	80	12.9	-0.014
Chamaemelum nobile	213	42.5	-0.005									
Chamerion angustifolium	36	2.1	-0.078	317	1.0	0.010	41	2.0	0.012	123	4.5	-0.018
Chenopodium album	65	10.1	-0.006	248	2.8	0.023	24	1.9	0.007	46	2.9	-0.013
Chenopodium rubrum	260	10.1	0.002	186	4.3	0.091						
Chrysosplenium alternifolium	353	10.7	-0.017	313	18.8	-0.017	11	40.4	-0.011	98	33.5	-0.007
Chrysosplenium oppositifolium	230	16.5	0.001	311	22.8	-0.102	42	4.0	-0.011	305	1.0	-0.022
Cicendia filiformis	226	32.9	0.002									
Circaea lutetiana	215	4.5	0.005	288	4.2	0.045	85	1.8	-0.118	177	19.8	-0.019

Cirsium acaule	163	9.6	-0.032									
Cirsium arvense	47	0.8	0.003	355	1.1	0.002	6	1.6	0.001	78	2.9	0.002
Cirsium dissectum	262	13.7	0.002									
Cirsium eriophorum	285	21.1	-0.005	294	6.5	-0.020						
Cirsium heterophyllum				331	41.6	0.030				358	16.1	0.006
Cirsium palustre	245	2.5	-0.046	314	14.8	-0.146	29	1.2	-0.020	354	1.7	-0.006
Cirsium vulgare	51	0.7	0.007	339	0.2	0.023	35	1.0	-0.045	6	5.5	0.044
Cladium mariscus	56	79.8	0.011							298	1.4	-0.005
Clematis vitalba	148	2.2	0.030	81	1.5	0.027	158	12.6	-0.044			
Clinopodium acinos	69	12.6	-0.031									
Clinopodium ascendens	56	2.3	0.013									
Clinopodium calamintha	257	84.3	-0.001									
Clinopodium vulgare	134	5.2	-0.035	310	4.7	0.010	36	0.9	-0.105	114	19.4	-0.001
Cochlearia danica	40	72.4	0.156	112	4.5	0.164	356	5.4	0.121	60	16.1	0.021
Coeloglossum viride	209	28.1	-0.012	323	3.0	-0.001				6	0.5	0.002
Colchicum autumnale	297	20.4	-0.011									
Comarum palustre	42	53.5	0.011	329	5.0	-0.040	28	29.5	0.092	323	1.0	-0.027
Conopodium majus	211	1.4	-0.043	322	16.4	-0.197	38	2.4	-0.091	248	1.8	-0.064
Convallaria majalis	66	18.1	0.005	165	0.7	-0.007						
Convolvulus arvensis	81	17.6	-0.012	125	8.5	0.073	198	5.0	-0.107	21	6.5	-0.020
Corallorhiza trifida										316	34.8	0.002
Cornus sanguinea	103	9.4	0.038	327	1.5	0.137	293	33.1	-0.028			
Cornus suecica										155	3.1	-0.008
Corylus avellana	74	4.9	0.058	175	1.0	0.094	140	4.2	-0.038	109	2.6	0.057
Crassula tillaea	72	62.2	0.014									
Crataegus laevigata	253	26.9	-0.017	155	21.4	0.032						
Crataegus monogyna	49	0.1	-0.005	338	3.1	0.025	59	0.1	-0.008	73	1.9	-0.031
Crepis biennis	101	1.6	-0.015	285	6.4	0.016						
Crepis capillaris	110	2.1	0.030	176	4.4	0.044	332	2.1	0.003	29	4.8	0.016
Crepis paludosa				148	5.5	-0.093	15	44.7	-0.024	177	12.0	-0.079
Cruciata laevipes	176	9.3	-0.021	241	4.1	-0.024	216	16.3	-0.128	355	19.4	-0.005
Cryptogramma crispa							25	2.6	0.015	180	31.4	0.006
Cuscuta epithymum	196	12.9	-0.003									
Cuscuta europaea	300	16.3	0.003									
Cynoglossum officinale	102	23.2	0.010	102	4.9	0.010						
Cynosurus cristatus	52	0.5	-0.048	324	3.6	-0.127	234	2.2	0.012	341	3.9	0.001
Cyperus longus	246	3.7	0.007									
Cystopteris fragilis	291	56.1	0.009	126	8.7	-0.026				135	6.4	-0.035
Cytisus scoparius	92	9.3	-0.022	340	5.1	-0.074	47	5.6	0.041	323	16.0	0.041
Dactylis glomerata	352	0.1	0.003	123	0.5	0.015	319	0.0	0.011	22	3.9	0.003
Dactylorhiza fuchsii	112	6.2	-0.020	308	11.8	-0.066	110	1.4	-0.066	162	7.2	-0.036
Dactylorhiza incarnata	60	14.5	-0.006	348	2.3	-0.012	238	24.9	0.032	327	10.1	0.026
Dactylorhiza maculata	244	49.4	-0.003	203	10.4	-0.023	247	10.8	0.177	321	7.4	0.150
Dactylorhiza praetermissa	33	10.3	-0.020	141	2.5	0.054	78	45.0	0.097			
Dactylorhiza purpurella				337	46.8	0.003	50	3.9	0.019	10	11.7	0.037
Danthonia decumbens	243	29.9	0.001	135	5.5	-0.096	280	3.8	0.130	300	3.2	0.072
Daphne laureola	285	1.9	0.019	292	3.9	0.005				327	95.5	0.006
Daucus carota	106	5.8	-0.060	16	1.2	0.063	207	8.4	0.074	285	5.3	0.013
Deschampsia cespitosa	239	2.9	-0.027	330	3.8	-0.079	26	1.8	-0.039	118	1.1	-0.002
Deschampsia flexuosa	312	17.8	0.009	312	25.9	-0.156	29	3.3	-0.125	318	1.4	-0.031

Deschampsia setacea										219	12.6	-0.003
Dianthus deltoides	9	68.7	-0.002							324	20.8	0.005
Digitalis purpurea	77	14.7	0.053	303	11.9	-0.075	109	1.4	-0.010	205	4.1	0.017
Diphasiastrum alpinum							357	27.5	0.022	278	8.4	0.009
Dipsacus pilosus	260	2.5	-0.013									
Draba incana										348	5.0	0.001
Draba muralis	271	16.9	0.004									
Drosera anglica										335	34.7	-0.021
Drosera intermedia	148	28.5	-0.001							237	7.3	-0.017
Drosera rotundifolia	321	16.9	-0.012	139	48.8	-0.055	316	9.4	-0.023	217	3.4	0.010
Dryas octopetala										256	16.7	-0.011
Dryopteris aemula	62	49.9	0.005							251	3.3	0.012
Dryopteris carthusiana	80	11.5	0.022	330	7.0	0.055	23	17.6	0.024	72	12.2	-0.025
Dryopteris dilatata	63	4.9	0.067	313	19.1	-0.085	344	1.9	0.062	143	2.1	-0.021
Dryopteris expansa										220	3.1	-0.014
Dryopteris filix-mas	58	2.1	0.019	313	6.7	-0.046	60	1.1	-0.024	124	5.5	-0.072
Dryopteris oreades										220	17.0	0.003
Echium vulgare	75	23.1	0.009	30	5.2	0.044	212	12.3	0.005	338	4.9	0.001
Elatine hexandra										28	19.0	-0.002
Eleocharis acicularis	91	18.5	-0.003							253	24.2	-0.003
Eleocharis multicaulis	281	37.3	0.014	136	72.1	-0.015	249	3.3	0.109	205	6.7	0.022
Eleocharis palustris	273	2.6	0.017	332	6.6	0.011	225	2.4	0.139	206	0.8	-0.024
Eleocharis quinqueflora	38	7.9	0.007	144	10.9	-0.014	231	22.7	-0.011	163	5.0	0.028
Eleocharis uniglumis	245	11.0	0.000							282	7.8	0.008
Eleogiton fluitans	242	57.2	0.009				254	3.4	0.108	254	3.0	-0.015
Elymus caninus	18	11.3	-0.018	305	2.6	-0.042	158	1.8	-0.133	122	4.4	-0.024
Elytrigia atherica	73	7.5	0.001	345	5.7	0.003	180	3.3	0.045			
Elytrigia repens	65	11.4	-0.041	330	10.0	0.044	348	1.0	-0.051	314	16.1	0.002
Empetrum nigrum				282	21.8	-0.022	339	15.7	0.057	10	6.6	0.021
Epilobium alsinifolium										228	24.9	-0.011
Epilobium anagallidifolium										70	1.6	0.000
Epilobium hirsutum	53	6.1	0.049	324	7.5	0.089	200	7.7	0.172	55	6.0	0.020
Epilobium lanceolatum	238	9.2	0.020									
Epilobium montanum	193	2.1	-0.020	325	1.2	-0.071	46	2.2	-0.028	344	6.2	0.012
Epilobium obscurum	238	19.8	0.098	132	1.2	0.145	324	2.6	0.039	162	2.8	0.027
Epilobium palustre	277	29.7	-0.006	103	2.8	-0.193	53	3.0	-0.103	344	2.0	0.012
Epilobium parviflorum	97	4.6	0.253	151	6.5	0.208	291	1.8	0.060	115	3.4	-0.033
Epilobium roseum	258	11.3	-0.020	238	8.4	0.019				122	17.0	0.000
Epilobium tetragonum	83	1.7	0.195	133	2.6	0.108	164	3.3	-0.072			
Epipactis helleborine	164	2.8	-0.010	325	5.1	0.005	213	22.6	-0.020	11	51.9	-0.001
Epipactis palustris	53	45.4	0.008									
Epipactis phyllanthes	88	2.0	-0.001									
Epipactis purpurata	80	13.7	-0.020									
Equisetum arvense	63	7.2	0.016	307	2.7	0.028	334	1.2	-0.085	2	3.7	-0.016
Equisetum fluviatile	47	7.4	0.022	333	3.0	-0.034	12	0.8	0.016	15	1.4	-0.026
Equisetum hyemale				349	22.9	0.009				353	3.8	-0.005
Equisetum palustre	56	11.4	-0.036	313	3.0	-0.143	215	2.4	-0.009	11	6.9	-0.014
Equisetum pratense										67	1.5	0.002
Equisetum sylvaticum	292	40.6	-0.009	317	34.8	-0.112	173	11.0	0.010	181	7.4	0.014
Equisetum telmateia	73	8.5	0.008	351	6.2	-0.034	207	25.2	0.002	65	15.7	-0.005

Equisetum variegatum										55	4.0	0.002
Erica ciliaris	178	53.0	-0.002									
Erica cinerea	236	24.4	-0.027	307	10.6	-0.036	340	12.4	0.117	312	3.2	0.016
Erica tetralix	341	8.1	-0.023	225	8.9	-0.041	2	12.1	0.027	193	5.5	0.011
Erigeron acris	78	14.3	0.020	115	6.3	0.039	150	13.2	0.062			
Eriophorum angustifolium	244	41.1	-0.006	138	30.0	-0.102	241	3.1	0.037	186	3.6	-0.018
Eriophorum latifolium				234	7.9	-0.012				139	9.0	0.004
Eriophorum vaginatum	204	43.0	-0.002	146	43.8	-0.056	319	7.8	0.031	280	3.4	-0.008
Erophila glabrescens	195	14.9	0.022							158	1.5	0.030
Euonymus europaeus	90	6.6	0.061	308	21.5	0.044	258	18.4	-0.030			
Eupatorium cannabinum	78	8.2	0.009	229	6.4	0.004	194	5.2	0.000	246	13.4	-0.014
Euphorbia amygdaloides	206	4.8	0.010				135	27.1	0.009			
Euphorbia stricta	270	23.4	0.005									
Euphrasia arctica										80	4.0	-0.013
Fagus sylvatica	85	4.6	0.028	84	2.3	0.047	139	2.8	-0.117	132	10.7	0.015
Festuca altissima										117	30.6	-0.017
Ficaria verna	161	2.3	0.043	310	8.3	-0.058	231	1.9	-0.017	4	10.6	-0.051
Filago minima	6	1.3	0.014	110	5.3	0.019				203	24.6	0.028
Filago vulgaris	267	20.0	0.089	326	23.3	0.043						
Filipendula ulmaria	103	0.3	-0.006	311	3.4	-0.025	184	1.5	-0.066	174	1.0	-0.003
Filipendula vulgaris	260	3.3	-0.011	294	23.9	-0.002						
Fragaria vesca	211	6.5	0.001	303	3.8	-0.085	34	1.4	-0.159	179	7.2	-0.053
Frangula alnus	201	12.2	0.014									
Fraxinus excelsior	224	1.8	0.048	332	2.6	0.057	200	0.7	-0.073	182	1.9	0.002
Fritillaria meleagris	166	11.6	0.002									
Fumaria bastardii	245	8.9	-0.004				150	36.5	0.037	153	3.1	0.007
Fumaria capreolata	36	14.2	0.008	5	29.5	-0.006	147	1.3	0.054	19	50.4	0.003
Fumaria muralis	236	56.0	0.030	252	5.4	0.052	289	4.4	0.082	108	7.3	-0.008
Fumaria occidentalis	252	15.5	-0.007									
Fumaria purpurea										39	14.1	0.012
Gagea lutea				1	50.8	0.000						
Galium album	212	7.9	-0.006	337	22.5	0.053	126	1.1	-0.005	76	4.0	-0.019
Galium aparine	49	0.6	0.002	355	2.4	0.024	16	1.5	0.022	355	7.1	0.064
Galium boreale										20	8.0	-0.026
Galium odoratum	240	16.5	-0.031	287	16.1	-0.036	178	14.8	-0.041	112	0.8	-0.026
Galium palustre	131	1.5	0.006	288	9.9	-0.134	285	1.3	0.015	192	3.9	-0.012
Galium parisiense	233	12.6	0.003									
Galium saxatile	296	2.3	-0.021	321	33.4	-0.165	22	5.7	-0.018	319	2.4	-0.022
Galium uliginosum	57	3.4	0.005	328	7.4	-0.014	267	0.9	0.000	139	7.7	-0.008
Galium verum	41	4.1	0.028	271	6.4	0.083	16	12.4	0.103	23	5.8	-0.042
Gaudinia fragilis	205	66.8	0.007									
Genista anglica	234	36.8	-0.009				360	3.7	0.022	299	3.7	-0.012
Genista tinctoria	147	15.7	-0.052	323	19.0	0.005						
Gentiana pneumonanthe	249	10.8	-0.004									
Gentianella amarella	214	10.1	-0.029	300	10.4	-0.012				34	8.6	0.003
Gentianella anglica	334	11.4	-0.010									
Gentianella campestris										309	2.6	0.030
Gentianella germanica	57	2.0	-0.014									
Geranium columbinum	237	37.6	-0.005				168	1.5	0.019			
Geranium lucidum	246	18.9	0.092	177	0.3	0.100	175	18.4	0.014	110	8.8	0.005

Geranium molle	67	5.9	0.065	138	11.7	0.112	207	0.3	-0.007	110	5.0	-0.029
Geranium pratense	8	11.5	-0.039	202	9.6	0.051	237	8.4	0.007	17	2.9	-0.012
Geranium purpureum	67	46.6	0.000									
Geranium pusillum	49	5.2	0.127	148	5.5	0.138				67	24.7	0.007
Geranium robertianum	68	2.9	0.042	301	1.4	0.078	303	0.6	-0.008	167	4.8	-0.001
Geranium rotundifolium	130	2.2	0.059									
Geranium sanguineum	310	2.1	0.001	343	9.8	0.006	332	2.5	0.058	24	9.4	0.001
Geranium sylvaticum				353	9.1	0.002				153	15.0	-0.028
Geum rivale	50	17.2	-0.018	157	12.5	-0.037	5	1.2	-0.036	177	3.8	-0.039
Geum urbanum	84	2.6	0.061	245	2.1	0.151	2	1.2	-0.070	130	4.9	-0.014
Glechoma hederacea	95	0.8	0.013	322	8.3	0.023	346	2.8	-0.011	358	4.4	-0.016
Glyceria declinata	251	11.1	-0.013	297	12.0	-0.014	169	9.8	-0.026	164	4.9	-0.037
Glyceria fluitans	230	5.8	-0.053	288	1.8	-0.180	350	0.6	-0.011	133	2.5	-0.085
Glyceria maxima	42	6.8	0.004	149	16.2	0.133	309	9.5	-0.002	97	13.3	0.003
Glyceria notata	52	10.4	-0.049	212	2.2	0.028				127	7.1	-0.024
Gnaphalium supinum										166	2.3	-0.003
Gnaphalium sylvaticum	16	4.0	-0.009							0	9.5	0.031
Gnaphalium uliginosum	84	10.2	0.015	223	6.1	-0.003	351	2.4	-0.018	357	1.3	0.073
Goodyera repens										6	80.0	0.035
Groenlandia densa	33	3.4	-0.004	140	55.9	0.005						
Gymnocarpium dryopteris				327	17.2	-0.011				115	4.2	-0.060
Hammarbya paludosa										334	3.8	-0.007
Hedera helix	285	0.0	0.029	86	1.4	0.032	113	0.9	-0.055	206	1.5	-0.004
Helianthemum nummularium	265	13.5	-0.001	299	24.7	0.015	113	5.9	-0.020	31	33.5	0.018
Helleborus foetidus	222	2.0	0.049	46	5.0	0.014						
Helleborus viridis	224	1.8	-0.002									
Heracleum sphondylium	86	0.6	0.003	348	2.2	0.027	1	0.2	-0.042	349	6.2	-0.009
Herminium monorchis	295	8.2	0.000									
Hippocrepis comosa	210	16.8	-0.017									
Hippophae rhamnoides	64	23.8	0.026	30	6.8	0.035	98	1.8	0.054	8	28.0	0.009
Hippuris vulgaris	77	1.6	-0.005	64	2.3	0.023				54	6.1	-0.030
Holcus lanatus	225	0.2	0.007	134	0.6	-0.001	336	0.0	0.034	23	1.6	0.023
Holcus mollis	261	10.3	-0.035	306	15.5	-0.146	17	1.6	-0.173	166	2.0	-0.033
Hordelymus europaeus	19	21.2	-0.022									
Hordeum marinum	233	85.1	0.009									
Hordeum secalinum	239	24.4	-0.022									
Hottonia palustris	42	43.1	-0.003	162	21.6	0.013						
Humulus lupulus	92	6.5	0.034	322	0.6	0.052	288	17.5	-0.091	145	1.9	-0.008
Huperzia selago							22	2.2	-0.004	314	10.9	0.004
Hyacinthoides non-scripta	244	7.6	-0.040	316	11.6	-0.092	163	1.2	-0.018	251	5.9	-0.034
Hydrocharis morsus-ranae	80	60.8	0.013									
Hydrocotyle vulgaris	76	6.3	-0.023	159	5.3	-0.073	98	7.7	-0.014	47	0.6	-0.022
Hymenophyllum tunbrigense	60	93.4	0.001							203	5.2	-0.004
Hymenophyllum wilsonii							63	1.2	-0.002	290	14.2	-0.017
Hypericum androsaemum	219	17.0	0.123	94	2.1	0.069	111	2.1	0.031	258	7.3	0.056
Hypericum elodes	232	66.1	-0.011				310	4.7	0.062	193	7.0	0.002
Hypericum hirsutum	236	1.4	-0.014	298	28.0	0.011	116	3.6	-0.103	91	11.3	-0.014
Hypericum humifusum	209	11.5	0.002	158	2.6	-0.033	333	5.6	-0.062	179	3.6	0.013
Hypericum maculatum	67	8.5	-0.015	122	34.1	0.025	61	6.8	-0.145	111	7.7	0.001
Hypericum montanum	260	8.5	-0.002									

Hypericum perforatum	71	1.8	0.063	302	2.1	0.040	276	4.4	-0.035	117	22.7	-0.033
Hypericum pulchrum	231	49.1	-0.038	313	8.8	-0.047	15	3.9	0.015	316	5.5	0.037
Hypericum tetrapterum	108	1.9	0.041	214	2.3	-0.027	238	1.0	-0.042	208	7.3	-0.030
Hypericum undulatum	63	205.9	0.001									
Hypochaeris glabra	62	59.3	0.016									
Hypochaeris radicata	227	3.2	0.002	280	1.9	-0.093	50	2.1	0.066	352	4.1	0.026
Hypopitys monotropa	36	1.3	0.004									
Ilex aquifolium	76	6.4	0.044	53	1.7	0.113	86	3.8	-0.064	157	5.9	-0.012
Illecebrum verticillatum	164	34.3	0.001									
Inula conyzae	213	19.3	-0.001	317	21.8	0.016	160	8.2	0.054			
Iris foetidissima	154	10.3	0.182	198	23.3	0.041	358	2.8	0.051			
Iris pseudacorus	54	4.5	0.076	182	1.6	0.196	219	5.0	0.081	270	2.8	0.001
Isoetes echinospora										177	1.1	-0.008
Isoetes lacustris										233	8.2	-0.003
Isolepis cernua	228	3.7	0.008				298	1.2	0.085	259	18.2	0.018
Isolepis setacea	255	11.2	0.007	294	1.7	-0.027	312	2.7	0.070	223	1.2	0.047
Jasione montana	237	123.1	-0.054	3	4.1	-0.018	95	3.5	-0.056	194	5.3	-0.011
Juncus acutiflorus	245	12.8	0.034	324	6.6	-0.018	282	3.8	0.025	191	9.7	-0.078
Juncus alpinoarticulatus										117	4.0	0.003
Juncus articulatus	141	2.0	-0.034	318	1.8	-0.165	167	0.2	0.037	338	1.0	0.046
Juncus bulbosus	228	27.5	-0.015	147	27.4	-0.131	203	0.7	0.004	319	5.8	0.030
Juncus compressus	46	27.5	0.000									
Juncus conglomeratus	181	6.9	-0.026	309	6.2	-0.050	326	3.4	0.105	7	3.7	0.027
Juncus effusus	144	0.3	0.004	300	6.5	-0.078	11	3.4	0.000	222	0.8	0.015
Juncus inflexus	63	12.7	0.085	317	5.1	0.117	228	9.4	0.012	143	38.4	-0.047
Juncus squarrosus	264	29.4	0.006	330	12.0	-0.047	337	9.5	-0.018	259	1.3	0.015
Juncus subnodulosus	39	23.7	0.023	184	2.2	0.011	67	27.6	-0.003			
Juncus trifidus										162	17.3	-0.027
Juncus triglumis										161	21.8	-0.019
Juniperus communis	176	27.6	-0.005	145	58.8	0.005				79	1.5	0.046
Kalmia procumbens										66	1.8	-0.004
Knautia arvensis	78	4.9	-0.093	290	6.4	0.015	277	4.9	-0.024	279	23.7	-0.003
Koeleria macrantha	265	6.2	-0.024	323	2.1	-0.010	241	15.9	-0.007	5	19.9	0.019
Lactuca virosa	266	44.9	0.078	324	90.1	0.064						
Lamiastrum galeobdolon	108	7.0	0.093	338	9.5	0.107	294	16.0	-0.040	35	6.8	0.036
Lapsana communis	59	2.3	0.045	324	3.9	0.072	30	7.0	-0.122	169	1.2	-0.015
Lathraea squamaria	228	1.7	-0.004	284	8.1	-0.006						
Lathyrus aphaca	145	14.6	0.003									
Lathyrus linifolius	237	39.0	-0.003	321	16.4	-0.130	169	1.8	-0.101	3	10.5	-0.039
Lathyrus nissolia	218	10.7	0.017									
Lathyrus pratensis	80	1.3	-0.031	329	1.1	-0.003	6	0.7	-0.004	32	3.2	0.008
Lathyrus sylvestris	230	4.3	-0.002									
Lemna gibba	269	15.6	0.000	127	82.2	0.007						
Lemna minor	41	5.2	-0.003	165	3.5	0.167	170	6.4	0.119	118	9.5	-0.004
Lemna trisulca	53	20.5	0.035	153	3.5	0.063	232	24.8	0.017	4	28.4	-0.001
Leontodon hispidus	105	2.4	-0.028	330	7.6	-0.045	176	6.3	-0.201	152	53.8	-0.017
Leontodon saxatilis	180	11.5	0.055	271	0.7	-0.003	211	5.8	0.034	184	7.2	-0.008
Lepidium heterophyllum	229	31.9	-0.017				339	12.3	0.009	106	5.8	-0.015
Lepidium latifolium	251	135.9	0.003									
Leucanthemum vulgare	77	1.2	0.022	315	1.9	-0.001	228	1.7	0.011	360	10.4	-0.003

Leucojum aestivum	335	31.6	0.038									
Ligustrum vulgare	84	1.9	0.027	121	4.9	0.135	225	4.9	-0.094	134	16.4	-0.024
Linaria vulgaris	70	9.6	-0.111	175	9.6	-0.001	179	9.6	-0.054	356	37.5	-0.015
Linnaea borealis										37	6.5	0.009
Linum bienne	195	3.3	-0.002									
Linum catharticum	223	3.0	-0.034	277	5.4	-0.071	81	1.3	-0.005	249	1.6	0.009
Lithospermum officinale	60	8.8	0.019									
Littorella uniflora	259	39.9	0.005	130	1.9	-0.010	180	0.2	0.046	145	4.3	-0.033
Lobelia dortmanna										316	21.1	-0.023
Lolium perenne	50	0.7	0.017	345	0.8	0.008	14	0.3	-0.015	34	5.0	0.037
Lonicera periclymenum	66	4.0	0.034	312	4.4	-0.055	37	4.0	-0.009	176	1.7	0.013
Lotus corniculatus	199	1.2	-0.012	331	2.5	-0.091	160	0.2	0.034	355	3.4	-0.013
Lotus pedunculatus	237	6.2	-0.037	243	8.0	-0.077	324	1.9	0.020	172	16.0	-0.106
Lotus subbiflorus	61	36.2	-0.001									
Lotus tenuis	310	22.6	-0.001									
Luronium natans							295	39.9	-0.004			
Luzula campestris	224	1.5	0.024	313	7.2	-0.151	332	1.9	0.014	155	1.4	-0.023
Luzula forsteri	190	44.6	0.011									
Luzula multiflora	63	3.5	0.008	322	21.8	-0.132	297	1.2	-0.057	342	5.5	-0.023
Luzula pilosa	226	18.5	-0.044	325	17.0	-0.059	186	2.8	-0.107	347	2.8	0.009
Luzula spicata										322	29.2	0.006
Luzula sylvatica	247	72.7	-0.003	330	39.5	-0.063	104	7.5	-0.029	148	4.6	-0.004
Lycopodiella inundata	161	30.5	0.001									
Lycopodium annotinum										210	46.9	-0.030
Lycopodium clavatum				339	45.3	-0.004	11	19.0	0.019	46	8.9	0.029
Lycopus europaeus	69	5.2	0.078	191	8.5	0.146	240	6.2	0.030	20	31.2	0.034
Lysimachia nemorum	237	20.8	0.014	315	25.1	-0.082	33	0.6	-0.118	219	5.3	-0.028
Lysimachia nummularia	82	7.7	0.029	37	1.5	0.074	163	2.9	-0.173	152	27.6	-0.003
Lysimachia thyrsiflora										161	12.5	-0.004
Lysimachia vulgaris	80	19.3	0.008	340	3.1	0.046	171	1.3	0.015	20	8.6	-0.005
Lythrum portula	232	23.9	0.008	84	1.4	0.000	103	10.3	0.090	173	8.6	0.010
Lythrum salicaria	181	2.4	0.011	188	8.2	0.099	174	5.1	0.128	186	5.2	-0.002
Malva arborea	61	20.9	0.021				358	1.3	0.075			
Malva moschata	109	8.7	0.080	12	1.0	0.107	296	14.8	-0.073	48	6.1	0.008
Marrubium vulgare	83	7.1	0.009									
Meconopsis cambrica	172	3.5	0.041	52	1.7	0.082	12	8.6	0.079	1	6.8	0.011
Medicago arabica	138	15.4	0.093									
Medicago lupulina	79	6.3	0.022	309	2.6	0.109	254	7.2	0.024	94	16.3	0.001
Medicago minima	238	56.4	-0.006									
Medicago polymorpha	174	14.5	0.011									
Medicago sativa	71	6.9	-0.037	64	6.6	0.009						
Melampyrum pratense	225	33.2	-0.042	162	16.8	-0.030	128	7.9	-0.040	3	1.9	-0.076
Melica nutans										49	1.6	-0.022
Melica uniflora	211	11.5	0.025	239	6.1	-0.049	194	2.2	-0.107	71	5.8	-0.026
Melittis melissophyllum	243	61.5	-0.001									
Mentha aquatica	83	3.6	-0.001	293	2.8	-0.069	241	4.3	-0.026	183	5.9	-0.034
Mentha arvensis	176	2.6	-0.034	155	0.6	0.024	161	2.9	-0.095	293	1.0	-0.033
Mentha pulegium	166	7.8	0.004									
Mentha suaveolens	233	5.7	0.002									
Menyanthes trifoliata	59	8.1	0.011	120	7.9	-0.049	148	16.4	0.017	314	4.1	-0.011

Mercurialis perennis	300	1.8	0.020	314	8.7	-0.016	76	7.7	-0.106	101	5.5	-0.034
Meum athamanticum										332	36.6	-0.007
Milium effusum	204	4.8	-0.061	193	30.4	-0.008	208	34.8	-0.027	106	6.4	-0.023
Minuartia sedoides										266	9.2	0.002
Minuartia verna				317	37.3	0.014						
Moehringia trinervia	259	3.9	-0.012	154	9.3	0.013	27	8.2	-0.052	113	22.2	-0.044
Moenchia erecta	178	28.9	0.002									
Molinia caerulea	335	9.9	-0.001	308	29.1	-0.043	24	3.6	-0.015	240	2.0	0.008
Montia fontana	206	12.3	0.017	137	28.7	-0.102	77	0.8	0.062	217	1.7	0.002
Muscari neglectum	253	68.3	0.007									
Mycelis muralis	59	8.5	-0.002	70	1.5	-0.024	19	7.1	-0.188	106	4.6	0.010
Myosotis discolor	93	14.2	0.017	313	2.4	-0.022	355	2.5	0.005	81	5.4	0.035
Myosotis laxa	43	17.4	0.006	297	0.4	0.082	195	2.5	0.033	143	2.6	-0.003
Myosotis ramosissima	177	2.8	0.038	106	2.3	0.015	179	1.0	-0.003	81	22.3	0.013
Myosotis scorpioides	43	2.2	-0.044	312	4.6	0.017	259	2.1	-0.151	130	12.9	-0.063
Myosotis secunda	178	4.0	-0.011	320	54.6	-0.026	352	4.4	-0.048	223	1.9	0.009
Myosotis stolonifera				166	16.1	-0.008						
Myosotis sylvatica	69	9.4	0.107	277	5.4	0.102	91	1.6	-0.042	106	11.3	-0.013
Myosoton aquaticum	87	6.2	-0.039	331	15.6	-0.006						
Myosurus minimus	246	41.9	0.003									
Myrica gale	246	9.9	-0.005	141	67.7	-0.037	334	32.4	0.002	341	6.1	0.003
Myriophyllum alterniflorum	220	67.0	-0.002	154	8.3	-0.029	186	36.0	-0.001	308	2.9	-0.031
Myriophyllum spicatum	229	4.6	-0.011	113	8.1	0.049	267	7.5	0.053	101	13.4	-0.019
Myriophyllum verticillatum	35	5.2	0.002									
Nardus stricta	253	35.9	-0.011	21	1.0	-0.127	334	7.3	-0.091	161	6.4	-0.050
Narthecium ossifragum	276	39.8	-0.015	162	34.7	-0.028	179	11.0	-0.008	180	6.9	-0.003
Nasturtium microphyllum	68	38.0	0.004	298	3.0	-0.019				127	5.5	-0.021
Neotinea ustulata	163	22.2	0.001									
Neottia cordata				194	11.8	-0.009				130	2.7	0.010
Neottia nidus-avis	238	4.3	0.011							343	49.2	0.004
Neottia ovata	123	4.8	-0.033	253	9.7	0.003	161	8.3	0.000	39	2.6	0.002
Noccaea caerulescens				359	0.9	0.006						
Nuphar lutea	207	14.4	-0.013	173	16.2	0.051	242	30.9	0.013	351	28.7	0.004
Nuphar pumila										247	8.2	0.002
Nymphaea alba	200	2.3	0.032	111	0.6	0.054	298	20.1	0.035	272	6.5	-0.012
Nymphoides peltata	216	2.2	0.020	286	5.3	0.015						
Odontites vernus	61	6.4	-0.044	314	5.0	0.084	220	1.6	-0.019	173	4.2	-0.042
Oenanthe aquatica	75	11.1	-0.002	336	3.6	0.007						
Oenanthe crocata	230	59.9	0.073	304	27.7	0.044	326	0.6	-0.056	208	28.9	-0.004
Oenanthe fistulosa	80	3.1	-0.013	226	5.1	0.011	356	36.7	-0.016			
Oenanthe fluviatilis	250	69.8	-0.015									
Oenanthe lachenalii	238	31.8	-0.008				308	23.1	0.014	195	6.8	-0.009
Oenanthe pimpinelloides	208	70.6	0.025									
Oenanthe silaifolia	43	45.6	0.006									
Onobrychis viciifolia	58	4.1	-0.026									
Ononis repens	56	1.2	-0.034	13	11.3	0.015	221	16.1	0.025	54	11.6	-0.016
Ononis spinosa	240	32.2	-0.013	182	76.5	0.025						
Ophrys apifera	69	14.0	0.079	109	3.8	0.080	186	1.5	0.066			
Ophrys insectifera	146	16.1	-0.001									
Orchis anthropophora	83	23.5	-0.008									

Orchis mascula	216	16.9	-0.028	295	20.3	-0.039	227	11.2	-0.053	288	7.6	-0.006
Oreopteris limbosperma	260	73.4	-0.001	239	3.7	-0.030	5	14.5	-0.033	267	3.6	0.023
Origanum vulgare	172	10.9	0.032	295	14.7	0.022	228	4.3	0.036	300	14.0	0.009
Ornithogalum pyrenaicum	219	30.5	0.002									
Ornithopus perpusillus	323	2.5	0.018	121	3.2	0.036	171	13.5	0.122	68	1.5	-0.002
Orobanche alba										293	6.5	0.006
Orobanche elatior	119	14.7	-0.021									
Orobanche hederae	88	20.5	0.016				82	1.9	0.047			
Orobanche minor	71	5.9	0.002									
Orobanche rapum-genistae	253	31.0	-0.002									
Orthilia secunda										9	42.7	-0.063
Osmunda regalis	236	22.7	0.000	16	0.7	0.000	181	2.0	0.037	260	4.2	-0.013
Oxalis acetosella	256	11.6	-0.032	320	38.4	-0.179	55	4.3	-0.052	186	2.3	-0.003
Oxyria digyna										288	19.8	0.014
Parentucellia viscosa	191	0.3	-0.015									
Parietaria judaica	206	2.3	0.052	249	1.3	0.053	122	0.5	0.038			
Paris quadrifolia	33	1.3	-0.016	355	1.6	-0.001						
Parnassia palustris				153	6.3	-0.043				243	2.3	0.018
Pastinaca sativa	273	12.7	-0.058	292	8.7	-0.001	283	18.4	-0.012			
Pedicularis palustris	51	25.5	-0.010	329	50.0	-0.031	267	24.6	-0.044	327	9.3	0.045
Pedicularis sylvatica	235	68.7	-0.041	155	7.3	-0.089	294	5.3	-0.012	186	2.7	-0.003
Persicaria amphibia	220	5.9	0.013	163	8.3	0.205	344	22.6	0.050	53	8.4	-0.018
Persicaria bistorta	42	4.0	-0.029	326	3.8	-0.058	80	1.3	-0.060	29	8.2	-0.022
Persicaria hydropiper	214	14.9	0.009	217	13.9	0.013	345	3.2	0.003	194	19.4	-0.022
Persicaria lapathifolia	61	4.8	-0.017	161	5.1	-0.014	34	1.9	-0.040	135	5.5	-0.026
Persicaria maculosa	63	5.8	-0.038	333	6.5	-0.094	25	0.9	-0.047	289	2.5	-0.043
Persicaria minor	227	17.0	0.004									
Persicaria mitis	24	3.8	-0.002									
Persicaria vivipara										198	13.1	-0.063
Petasites hybridus	326	4.2	-0.061	323	12.0	0.039	239	3.0	-0.110	358	2.9	-0.015
Petroselinum segetum	273	21.2	0.005									
Phalaris arundinacea	326	3.1	-0.003	181	2.3	0.053	6	1.5	-0.118	311	2.1	0.001
Phegopteris connectilis				338	4.1	-0.035	53	2.5	-0.029	262	7.3	-0.032
Phragmites australis	263	6.1	0.052	142	11.7	0.192	342	1.9	0.138	250	1.6	-0.001
Phyteuma orbiculare	2	4.8	0.001									
Picris hieracioides	114	24.0	-0.015									
Pilosella officinarum	287	2.2	-0.003	344	12.6	0.161	233	0.4	-0.030	175	1.0	0.010
Pilularia globulifera	217	79.9	0.000									
Pimpinella major	355	6.5	0.006	153	16.9	0.009						
Pimpinella saxifraga	121	4.8	-0.087	313	7.7	-0.019	1	4.6	-0.088	208	4.2	-0.045
Pinguicula lusitanica	232	141.9	-0.003							290	14.8	0.030
Pinguicula vulgaris				160	15.6	-0.069	330	7.2	-0.044	177	8.0	-0.051
Pinus sylvestris	243	3.3	0.055	293	1.6	0.017	37	2.9	-0.069	89	5.5	0.049
Plantago coronopus	30	17.7	0.091	84	12.5	0.063	260	9.9	0.115			
Plantago lanceolata	29	0.4	0.005	118	1.4	0.016	13	0.3	-0.013	3	0.9	-0.020
Plantago major	75	0.6	0.017	335	0.1	0.021	18	0.5	-0.036	19	2.2	0.059
Plantago maritima	77	16.0	0.001	5	18.0	0.020	10	14.6	0.058	113	10.7	0.047
Plantago media	72	11.0	-0.062	179	3.5	-0.029						
Platanthera bifolia	232	33.1	0.000							316	8.5	-0.003
Platanthera chlorantha	231	16.1	-0.008	314	11.8	-0.002				309	12.2	0.003

Poa alpina										303	16.7	-0.009
Poa annua	44	0.0	0.023	162	3.5	-0.002	47	1.1	-0.016	6	0.9	0.054
Poa compressa	223	1.8	0.014	291	3.0	0.012						
Poa glauca										295	12.6	0.000
Poa infirma	177	39.1	0.018									
Poa nemoralis	244	11.3	-0.059	318	6.0	-0.003	112	1.9	-0.153	125	4.6	-0.008
Poa trivialis	64	1.4	0.032	167	1.1	-0.002	241	1.5	-0.136	112	6.0	-0.102
Polemonium caeruleum	248	6.1	-0.001	308	2.8	0.013				42	6.9	0.002
Polygala calcarea	4	20.4	-0.013									
Polygala serpyllifolia	229	26.4	-0.027	130	6.7	-0.084	341	8.0	-0.065	192	4.3	-0.002
Polygala vulgaris	219	13.6	-0.051	233	3.7	-0.077	300	8.7	-0.030	333	12.6	0.001
Polygonatum multiflorum	205	33.7	-0.003									
Polygonum boreale										19	16.1	0.010
Polypogon monspeliensis	95	15.6	0.022									
Polystichum aculeatum	256	4.2	0.003	322	20.2	0.007	231	9.7	-0.081	130	1.4	-0.029
Polystichum lonchitis										130	20.0	-0.001
Polystichum setiferum	235	46.1	0.070	259	12.4	0.048	226	8.2	0.008	174	3.5	0.001
Populus nigra	224	21.4	0.020	170	26.7	0.121	164	2.0	-0.012	133	2.2	-0.018
Populus tremula	103	2.7	0.022	180	1.8	0.037	200	5.0	-0.051	154	3.7	0.022
Potamogeton alpinus										228	8.0	-0.009
Potamogeton berchtoldii	338	1.3	0.012	357	1.1	0.013	296	5.5	0.060	157	26.7	-0.016
Potamogeton coloratus	250	65.5	-0.003									
Potamogeton crispus	217	1.3	-0.017	202	0.3	0.031	271	4.7	-0.040	318	8.0	-0.030
Potamogeton filiformis										43	14.2	-0.009
Potamogeton friesii	51	14.1	0.004									
Potamogeton gramineus										163	10.3	-0.025
Potamogeton lucens	59	7.6	0.000	48	1.0	0.009						
Potamogeton natans	79	2.6	0.016	309	1.2	0.046	194	26.7	0.043	138	4.0	-0.068
Potamogeton obtusifolius	39	12.4	0.003	220	2.8	0.006				158	25.9	-0.002
Potamogeton pectinatus	240	45.4	-0.033	159	10.0	0.073	242	38.9	0.022	136	5.9	-0.023
Potamogeton perfoliatus	218	6.3	-0.017	94	3.4	0.015				171	21.0	-0.026
Potamogeton polygonifolius	237	16.9	-0.002	142	8.8	-0.053	352	6.0	0.009	232	3.1	0.039
Potamogeton praelongus										40	2.5	0.000
Potamogeton pusillus	51	5.5	0.018	147	66.9	0.028				115	4.1	-0.013
Potamogeton trichoides	55	2.4	0.009									
Potentilla anglica	248	1.9	-0.035	321	6.0	0.002	158	4.4	-0.036	150	1.4	-0.019
Potentilla anserina	66	3.3	-0.027	306	2.3	0.064	21	1.3	0.049	2	2.6	-0.006
Potentilla argentea	246	48.3	0.004									
Potentilla crantzii										128	3.6	0.005
Potentilla erecta	263	13.5	-0.037	322	26.6	-0.352	14	7.6	-0.004	324	4.3	-0.026
Potentilla reptans	84	3.7	-0.017	7	5.3	0.062	183	2.6	-0.112	148	39.1	-0.032
Potentilla sterilis	217	4.0	-0.003	317	8.6	-0.072	358	3.0	-0.050	352	13.0	0.022
Poterium sanguisorba	210	8.0	-0.018	265	17.9	0.036	210	20.5	-0.056			
Primula farinosa				345	2.5	-0.012						
Primula scotica										3	9.1	0.003
Primula veris	73	9.7	-0.023	296	23.7	0.067	229	20.9	-0.014	102	5.2	-0.011
Primula vulgaris	111	4.6	0.045	333	15.7	-0.060	284	3.2	-0.149	305	1.2	-0.008
Prunella vulgaris	33	0.9	0.018	289	0.8	0.015	313	0.8	0.013	313	1.0	0.006
Prunus avium	65	5.4	0.138	145	5.0	0.189	237	9.7	-0.025	120	12.1	0.007
Prunus padus	52	29.4	0.037	241	3.4	-0.018	260	25.2	-0.037	113	10.8	0.009

Prunus spinosa	213	0.9	0.006	158	1.7	0.068	16	1.8	-0.047	156	9.5	-0.012
Pseudorchis albida										308	2.8	-0.015
Pteridium aquilinum	62	3.8	0.026	314	18.4	-0.200	23	0.6	-0.002	245	5.7	0.016
Puccinellia distans	273	51.5	0.054	305	7.9	0.200				93	17.1	0.055
Puccinellia fasciculata	298	46.7	0.006									
Pulicaria dysenterica	47	5.5	0.014	315	20.1	0.027	224	18.4	-0.022			
Pulmonaria longifolia	179	83.4	0.000									
Pyrola media										13	56.7	0.017
Pyrola minor										9	12.4	-0.008
Quercus petraea	253	23.3	-0.064	327	13.6	-0.115	140	6.4	-0.083	197	9.8	-0.054
Quercus robur	256	1.8	0.048	158	1.2	0.119	140	2.7	-0.096	145	5.0	0.011
Radiola linoides	230	117.2	-0.005				356	2.6	0.075	27	3.8	0.015
Ranunculus acris	246	1.7	-0.006	101	0.5	-0.032	20	2.1	-0.055	240	0.3	0.009
Ranunculus auricomus	176	7.4	-0.041	245	13.0	-0.017	240	33.5	-0.076	140	49.4	-0.027
Ranunculus bulbosus	253	3.4	0.041	96	1.8	0.031	264	3.7	-0.104	74	4.5	-0.001
Ranunculus flammula	228	6.5	-0.029	330	3.9	-0.190	318	2.1	0.075	286	1.9	0.009
Ranunculus hederaceus	247	38.8	0.007	118	1.8	-0.032	136	1.1	0.105	168	16.5	-0.017
Ranunculus lingua	52	2.7	0.021	354	2.1	0.034				146	32.1	0.001
Ranunculus omiophyllus	237	76.5	-0.031	119	7.5	-0.065	4	19.9	-0.034			
Ranunculus parviflorus	240	32.1	0.030									
Ranunculus repens	12	0.1	0.005	350	1.4	0.012	30	0.0	0.006	158	0.2	0.001
Ranunculus sardous	301	38.2	0.018									
Ranunculus sceleratus	28	1.6	-0.027	164	10.1	0.164	193	2.9	-0.002	22	8.2	0.002
Raphanus raphanistrum	80	6.7	0.057	195	5.8	-0.021	259	1.1	0.058	86	1.6	-0.012
Reseda lutea	103	5.5	-0.051	21	5.8	0.033				74	10.6	-0.008
Rhamnus cathartica	59	17.9	-0.014	96	0.8	0.040						
Rhinanthus minor	323	2.0	-0.040	321	10.9	-0.124	334	1.2	0.057	7	8.6	0.014
Rhynchospora alba	212	60.2	-0.004				215	1.5	0.005	292	15.4	0.000
Rhynchospora fusca	192	36.7	-0.002									
Ribes alpinum				211	4.5	0.003						
Ribes rubrum	183	3.2	0.019	281	1.8	0.112	21	2.6	-0.089	71	4.3	-0.001
Rorippa amphibia	228	21.3	-0.023	168	86.8	0.016						
Rorippa palustris	84	6.4	0.000	18	0.3	0.039	253	4.2	-0.058	41	8.4	-0.018
Rorippa sylvestris	94	10.0	-0.015	338	21.3	0.038	85	1.2	-0.062	134	42.4	-0.027
Rosa arvensis	242	4.6	0.003	181	26.9	0.108	288	3.2	-0.079			
Rosa caesia	16	9.1	0.005	198	12.6	0.021				336	14.9	0.112
Rosa micrantha	169	17.3	0.033									
Rosa obtusifolia	136	8.7	0.008									
Rosa sherardii	275	67.2	0.008	240	5.6	0.027	354	4.8	0.027	328	3.3	0.014
Rosa spinosissima	147	2.4	0.002	352	3.9	0.011	135	4.3	0.030	234	2.8	0.027
Rosa stylosa	174	14.9	0.016									
Rosa tomentosa	49	17.5	0.016									
Rubia peregrina	237	166.7	0.009				242	3.7	0.014			
Rubus caesius	70	42.8	0.052	87	7.8	0.055	205	15.8	-0.001			
Rubus chamaemorus				327	92.8	0.010				178	63.1	-0.072
Rubus idaeus	64	2.2	-0.063	336	6.3	-0.056	28	10.5	-0.135	121	4.1	-0.019
Rubus saxatilis										217	1.7	-0.031
Rumex acetosa	67	1.8	-0.007	324	1.6	-0.066	345	0.3	0.005	53	0.4	-0.016
Rumex acetosella	200	0.8	-0.055	270	3.1	-0.260	9	1.3	0.078	170	0.8	-0.093
Rumex conglomeratus	98	2.5	-0.092	160	9.1	0.137	308	1.7	0.011	4	36.4	-0.008

Rumex crispus	67	4.6	0.011	338	2.2	0.077	327	0.8	0.086	344	0.9	-0.032
Rumex hydrolapathum	42	17.2	0.010	198	13.6	0.054	27	30.4	-0.018			
Rumex longifolius				351	2.6	0.017				31	32.4	0.056
Rumex maritimus	232	71.6	-0.006	179	1.5	0.009						
Rumex obtusifolius	357	0.6	0.011	344	3.1	0.057	11	0.4	0.025	352	5.2	0.051
Rumex palustris	63	9.8	0.001									
Rumex pulcher	218	13.6	0.023									
Rumex sanguineus	101	3.3	0.105	0	1.3	0.194	99	1.6	-0.045	160	25.9	-0.027
Ruscus aculeatus	268	10.2	0.062									
Sagina apetala	93	5.9	0.086	360	0.7	0.081	358	7.0	0.093	63	4.2	0.033
Sagina nodosa	68	12.6	0.004	314	12.5	-0.014	63	1.0	0.056	1	3.2	0.014
Sagina procumbens	143	1.1	0.082	311	10.9	-0.024	1	2.4	0.090	348	5.4	0.017
Sagina subulata	65	129.1	0.008				310	53.5	0.004	305	7.9	0.032
Sagittaria sagittifolia	213	10.0	-0.009	161	63.4	0.029						
Salix aurita	253	54.7	-0.040	334	18.6	-0.043	273	1.7	-0.014	241	4.7	0.005
Salix caprea	80	8.9	-0.048	330	1.6	-0.083	154	5.9	-0.185	142	4.7	-0.042
Salix cinerea	73	1.8	0.050	196	0.8	0.049	144	0.3	0.043	135	4.3	-0.032
Salix herbacea										251	24.4	0.003
Salix lapponum										133	4.4	-0.005
Salix myrsinifolia										208	20.9	-0.009
Salix myrsinites										314	0.3	-0.010
Salix pentandra	55	41.3	0.008	6	0.8	-0.007				15	7.1	-0.007
Salix phylicifolia				10	3.5	0.013				176	3.7	-0.011
Salix purpurea	55	14.0	0.009	345	6.9	0.027				88	4.9	-0.006
Salix repens	144	17.1	-0.005	117	14.1	-0.038	256	2.7	0.081	181	9.8	-0.019
Salsola kali	57	97.5	0.003							334	3.0	-0.003
Salvia pratensis	358	2.3	0.001									
Salvia verbenaca	296	5.9	0.001									
Sambucus nigra	58	0.2	0.012	337	9.8	0.075	234	1.4	-0.022	309	19.2	0.038
Samolus valerandi	311	11.6	0.006	84	1.9	0.024	281	8.0	0.084	228	39.8	-0.003
Sanguisorba officinalis	320	26.2	0.002	334	13.3	-0.100	161	4.8	-0.076			
Sanicula europaea	232	23.4	-0.066	315	24.5	-0.037	39	7.7	-0.150	179	1.5	-0.038
Saussurea alpina										289	16.9	-0.013
Saxifraga aizoides										46	3.0	-0.033
Saxifraga granulata	51	17.7	-0.014	335	2.3	0.005				98	29.6	-0.012
Saxifraga hypnoides				26	1.1	0.001				186	16.4	0.002
Saxifraga nivalis										276	3.6	-0.001
Saxifraga oppositifolia										167	6.8	-0.017
Saxifraga stellaris										224	17.9	0.011
Saxifraga tridactylites	252	3.2	0.019	262	0.7	-0.008	181	11.6	0.038			
Scabiosa columbaria	212	10.6	-0.015	344	6.4	-0.002						
Schedonorus arundinaceus	114	1.2	0.095	169	2.5	0.192	308	3.3	0.012	68	4.2	-0.045
Schedonorus giganteus	178	1.8	-0.032	295	4.4	-0.019	18	2.3	-0.222	45	6.9	-0.034
Schedonorus pratensis	58	7.0	-0.081	66	1.7	-0.014	214	2.1	-0.101	125	7.3	-0.047
Schoenoplectus lacustris	306	3.3	-0.007	330	26.9	-0.005				175	6.4	-0.015
Schoenoplectus tabernaemon	271	78.3	0.007	28	0.6	0.044	227	7.6	0.069	29	3.5	-0.005
Schoenus nigricans	45	19.6	0.003				333	52.8	0.005	336	37.7	0.022
Scilla autumnalis	77	23.7	-0.001									
Scilla verna	181	5.8	-0.010				293	27.4	0.015	3	18.9	0.017
Scirpus sylvaticus	168	24.2	0.003	245	4.2	0.007	177	3.8	-0.018	156	67.1	-0.014

Scleranthus annuus	86	14.7	-0.017							155	19.8	-0.005
Scorzoneroides autumnalis	59	2.5	0.027	128	1.4	0.052	8	0.9	-0.020	350	2.3	-0.018
Scrophularia auriculata	289	0.7	0.048	263	3.2	0.169	194	2.2	-0.098			
Scrophularia nodosa	227	5.0	-0.011	306	12.4	-0.036	91	1.0	-0.050	177	4.5	-0.004
Scrophularia umbrosa	51	17.6	0.003							118	23.5	0.000
Scutellaria galericulata	216	3.9	0.009	216	11.0	0.024	37	25.2	0.040	228	2.6	0.043
Scutellaria minor	232	90.1	-0.004				47	9.4	0.051	273	24.1	0.014
Sedum acre	72	4.2	-0.010	15	2.5	0.033	189	8.2	-0.040			
Sedum anglicum	237	88.8	-0.011	3	3.3	0.001	50	1.5	0.022	259	8.6	0.031
Sedum forsterianum	80	3.5	-0.007				90	1.8	-0.080			
Sedum rosea										313	35.9	0.004
Sedum telephium	101	6.2	0.001	147	1.9	0.009	12	0.8	-0.047	341	17.1	-0.023
Sedum villosum										196	36.8	-0.004
Selaginella selaginoides				322	3.3	0.003	352	81.4	0.004	336	19.5	0.029
Senecio aquaticus	243	37.6	-0.063	69	2.0	-0.122	139	3.0	-0.018	338	5.2	-0.001
Senecio erucifolius	99	10.5	0.000	118	9.6	0.056	239	12.4	-0.026			
Senecio jacobaea	76	3.3	0.039	355	0.9	0.052	63	1.5	0.140	149	2.4	0.024
Senecio sylvaticus	75	11.0	-0.024	258	2.4	-0.004	165	7.2	0.089	96	7.2	0.034
Senecio vulgaris	70	5.4	-0.001	343	4.4	-0.007	296	0.6	-0.088	14	11.1	0.006
Serratula tinctoria	345	18.0	-0.030	336	11.0	0.010	334	15.1	-0.064			
Sesleria caerulea				310	53.3	0.009						
Sherardia arvensis	142	2.6	0.099	298	2.4	0.031	170	10.4	0.031	151	3.4	-0.001
Sibbaldia procumbens										157	1.1	-0.001
Sibthorpia europaea	243	75.0	-0.006									
Silaum silaus	105	10.2	-0.016	23	4.7	-0.011						
Silene acaulis										241	21.2	-0.004
Silene conica	56	92.0	-0.001									
Silene dioica	238	2.4	0.031	43	2.0	0.022	337	3.3	-0.083	47	8.8	0.033
Silene flos-cuculi	326	1.0	-0.054	302	9.8	-0.117	240	3.8	0.029	103	1.5	-0.007
Silene vulgaris	62	32.6	-0.037	17	10.9	-0.040	137	0.6	0.033	310	14.1	-0.022
Sison amomum	173	12.8	0.062									
Sium latifolium	62	67.0	0.005									
Solanum dulcamara	67	4.7	-0.051	177	13.6	0.170	320	7.8	0.036	117	11.0	-0.017
Solanum nigrum	86	4.3	0.116	22	0.7	0.118	11	8.7	0.143			
Solidago virgaurea	231	55.0	-0.031	323	15.2	-0.053	298	1.2	-0.003	162	7.6	-0.030
Sonchus arvensis	72	16.4	-0.049	320	14.2	0.050	321	5.6	-0.016	42	6.7	-0.002
Sonchus asper	72	2.5	0.027	312	3.8	0.105	183	1.9	0.011	345	10.5	0.122
Sonchus oleraceus	83	3.8	0.005	309	4.9	0.141	235	1.2	0.043	332	16.1	0.042
Sonchus palustris	57	112.1	0.008									
Sorbus aria	286	11.6	0.053	26	2.1	0.064	142	2.2	0.061	235	12.6	0.000
Sorbus aucuparia	71	7.6	0.074	312	8.2	-0.078	53	3.1	-0.071	224	1.9	-0.019
Sorbus devoniensis	67	151.2	0.002									
Sorbus torminalis	192	15.9	0.005									
Sparganium angustifolium							346	52.0	0.025	258	2.9	-0.005
Sparganium emersum	230	18.6	-0.015	182	26.8	0.045	169	19.3	-0.003	139	10.9	-0.014
Sparganium erectum	291	3.3	0.008	131	0.2	0.165	185	6.5	0.043	98	9.0	-0.004
Sparganium natans										298	4.0	-0.009
Spergula arvensis	76	8.5	-0.016	267	0.9	-0.157	316	2.9	0.037	311	1.4	-0.017
Spergularia marina	18	32.8	0.077	96	15.1	0.164	196	0.5	0.062	60	12.0	0.097
Spergularia rubra	235	10.0	0.032	208	16.8	0.003	329	1.7	0.064	46	6.7	0.033

Spiranthes spiralis	208	35.4	0.020				223	3.1	0.013			
Spirodela polyrhiza	24	12.2	0.015									
Stachys palustris	84	11.6	0.007	290	8.2	0.088	302	1.4	-0.052	172	7.5	-0.023
Stachys sylvatica	65	1.5	-0.014	340	1.9	0.006	2	1.0	-0.099	142	6.4	-0.038
Stellaria alsine	263	13.8	-0.026	311	24.2	-0.223	359	1.1	-0.080	330	0.9	-0.061
Stellaria graminea	166	0.4	-0.025	312	6.8	-0.118	13	1.3	0.016	125	5.1	-0.019
Stellaria holostea	63	4.2	-0.003	324	16.5	-0.171	23	11.9	-0.172	152	8.4	-0.064
Stellaria media	60	1.9	-0.010	17	7.0	0.035	351	0.9	-0.095	351	10.2	0.014
Stellaria neglecta	252	40.5	0.017				356	20.8	-0.111			
Stellaria nemorum				179	5.2	-0.001				139	26.5	-0.007
Stellaria pallida	80	27.7	0.059	118	17.7	0.035	211	2.3	0.041	49	7.1	0.013
Stellaria palustris	24	5.0	-0.003									
Stratiotes aloides	42	6.0	0.014									
Subularia aquatica										176	32.1	-0.019
Succisa pratensis	242	16.8	-0.035	320	23.7	-0.132	289	3.7	0.064	350	7.1	-0.024
Symphytum officinale	209	2.9	0.012	81	4.7	0.069				264	10.3	-0.036
Symphytum tuberosum	262	8.7	0.008							94	16.1	-0.023
Tamus communis	75	4.6	-0.018	178	6.4	0.001	231	12.3	-0.045			
Tanacetum vulgare	169	3.0	0.039	177	2.6	0.036	235	11.6	-0.086	5	33.9	0.022
Taxus baccata	221	9.5	0.081	134	1.8	0.194	151	1.2	-0.107	33	5.8	0.012
Teesdalia nudicaulis	77	29.9	-0.006							357	23.4	0.002
Teucrium scorodonia	53	6.6	0.004	318	24.6	-0.157	35	3.2	-0.034	239	1.4	-0.011
Thalictrum alpinum										332	25.3	0.013
Thalictrum flavum	305	8.3	-0.006	52	2.9	0.022						
Thalictrum minus	54	16.3	-0.003	326	12.8	0.019	20	1.2	0.001	7	2.5	-0.005
Thelypteris palustris	121	19.0	0.000									
Thesium humifusum	168	20.1	-0.009									
Thymus polytrichus	229	24.7	-0.048	331	22.4	-0.017	352	6.2	0.020	117	3.0	-0.023
Thymus pulegioides	352	0.9	-0.012									
Thyselium palustre	56	113.0	0.005									
Tilia cordata	193	17.9	0.046	293	8.2	0.036	196	35.9	-0.024			
Tilia platyphyllos	33	2.9	0.012	334	0.8	0.017				110	6.7	0.012
Tofieldia pusilla										130	9.9	-0.015
Torilis japonica	74	1.5	-0.032	291	3.1	0.135	24	3.9	-0.017	118	12.1	-0.012
Torilis nodosa	54	2.3	0.033									
Tragopogon pratensis	64	17.9	-0.040	94	1.9	0.102	312	22.3	-0.035	344	45.6	0.002
Trichomanes speciosum				188	19.1	0.003				267	2.6	0.001
Trientalis europaea										216	36.5	-0.063
Trifolium arvense	103	15.6	-0.004	119	2.8	0.014	327	16.2	0.033	54	10.0	0.011
Trifolium campestre	78	9.4	-0.028	33	0.8	0.060	176	4.2	0.000	45	12.3	-0.026
Trifolium dubium	87	1.2	0.019	139	1.2	0.090	344	0.5	0.094	23	7.0	0.020
Trifolium fragiferum	247	17.8	0.001									
Trifolium glomeratum	152	6.0	0.017									
Trifolium medium	268	5.6	-0.012	321	17.2	0.000	215	16.0	0.015	124	16.7	-0.061
Trifolium micranthum	268	7.8	0.078				306	0.8	0.105			
Trifolium ochroleucon	250	25.1	0.010									
Trifolium ornithopodioides	190	18.2	0.022									
Trifolium pratense	343	0.9	-0.018	32	0.5	-0.015	346	2.7	0.028	4	4.3	0.040
Trifolium repens	63	0.1	0.008	308	0.2	-0.001	19	0.1	-0.017	60	0.7	0.017
Trifolium scabrum	207	23.7	0.019									

Trifolium striatum	220	10.4	0.030	120	2.3	0.020	316	2.4	0.093			
Trifolium subterraneum	190	17.7	0.035									
Trifolium suffocatum	129	49.2	0.016									
Triglochin maritima	314	11.2	0.008	359	11.7	0.002	271	4.3	0.081	314	6.9	0.033
Triglochin palustris	44	18.5	-0.006	316	23.4	-0.059	296	4.4	0.026	299	1.8	-0.005
Trisetum flavescens	33	6.6	-0.019	336	2.0	0.017	171	4.5	-0.107	330	23.4	-0.002
Trollius europaeus				105	2.0	0.000				343	7.8	-0.013
Turritis glabra	56	48.3	0.001									
Tussilago farfara	51	7.6	-0.090	333	3.9	-0.105	127	4.8	-0.105	345	11.4	-0.035
Typha angustifolia	241	31.7	-0.001	161	3.9	0.043						
Typha latifolia	57	8.0	0.062	171	2.4	0.231	178	5.4	0.158	353	32.3	0.030
Ulex europaeus	77	4.5	0.012	327	9.0	-0.071	320	2.1	0.146	360	6.5	0.004
Ulex gallii	256	81.5	-0.044	240	23.7	0.005	354	2.7	0.088	2	24.5	-0.005
Ulex minor	158	41.0	-0.004									
Ulmus glabra	56	2.2	-0.009	113	3.0	-0.019	64	7.6	-0.233	62	3.4	-0.033
Ulmus procera	241	12.6	-0.101	208	1.3	0.001	223	1.1	-0.200			
Umbilicus rupestris	243	48.7	-0.033	224	3.5	-0.012	322	6.7	0.099	199	5.1	0.017
Urtica dioica	357	0.0	0.001	351	0.7	0.022	20	0.2	-0.015	89	0.9	0.011
Utricularia australis	228	14.9	-0.001							28	9.0	0.001
Utricularia intermedia										222	4.8	-0.032
Utricularia minor	94	12.7	-0.002							261	2.6	-0.020
Utricularia ochroleuca										182	1.5	-0.001
Utricularia stygia										155	17.1	0.001
Vaccinium microcarpum										19	52.2	0.012
Vaccinium myrtillus	256	72.3	-0.028	306	10.4	-0.078	33	5.3	-0.085	208	2.0	-0.044
Vaccinium oxycoccos				131	62.7	-0.017	332	13.0	0.014	347	63.7	-0.010
Vaccinium uliginosum										200	19.6	-0.020
Vaccinium vitis-idaea	346	5.9	-0.001	254	32.9	0.012	32	1.7	-0.004	24	6.5	-0.006
Valeriana dioica	52	28.2	-0.009	161	6.5	-0.082	96	3.6	-0.076	348	37.8	-0.007
Valeriana officinalis	268	23.4	-0.037	320	10.7	-0.119	139	0.8	-0.184	206	5.7	-0.028
Valerianella locusta	229	0.7	-0.020	65	0.9	0.009	331	3.8	0.004	165	1.3	-0.005
Verbascum lychnitis	251	6.1	-0.002									
Verbascum nigrum	127	14.9	0.012									
Verbascum pulverulentum	251	0.9	0.012									
Verbascum thapsus	97	5.5	0.045	128	2.1	0.131	177	2.9	0.082	40	8.8	0.011
Veronica anagallis-aquatica	251	12.4	-0.047	114	8.0	0.042				123	2.9	-0.026
Veronica arvensis	82	3.5	0.102	223	1.5	0.140	357	3.0	-0.019	41	5.6	0.035
Veronica beccabunga	216	2.2	-0.032	282	2.2	-0.057	39	3.7	-0.133	153	12.2	-0.059
Veronica catenata	282	4.2	-0.028	150	9.8	0.056						
Veronica chamaedrys	16	0.5	0.009	26	0.6	-0.009	5	2.2	-0.112	165	2.6	-0.048
Veronica montana	221	20.5	-0.003	273	9.3	0.029	312	4.7	-0.145	114	7.5	-0.028
Veronica officinalis	202	12.4	0.013	328	14.9	-0.132	12	3.1	0.058	146	2.5	-0.001
Veronica scutellata	13	4.3	0.017	324	23.0	-0.013	299	13.9	0.007	156	3.2	-0.031
Veronica serpyllifolia	216	4.6	0.088	319	12.4	-0.047	302	2.4	-0.075	176	1.4	-0.012
Viburnum lantana	125	3.7	0.025	138	2.3	0.030						
Viburnum opulus	72	6.1	0.025	332	2.8	0.073	323	4.1	-0.021	126	8.2	-0.012
Vicia cracca	64	3.7	-0.022	321	2.5	0.007	334	0.9	-0.074	18	9.9	-0.013
Vicia hirsuta	111	7.8	-0.066	223	3.9	0.105	244	15.9	-0.027	76	9.7	0.021
Vicia lathyroides	234	16.2	0.004							1	37.3	0.018
Vicia lutea	97	12.0	0.005									

Vicia orobus							207	6.1	-0.023	219	7.9	-0.002
Vicia parviflora	243	27.9	-0.004									
Vicia sativa	128	3.0	-0.086	154	10.0	0.079	291	1.3	-0.023	38	8.8	0.049
Vicia sepium	230	8.3	-0.054	101	1.2	-0.092	40	6.3	-0.173	332	2.1	-0.004
Vicia sylvatica	270	4.6	-0.015	191	24.7	-0.012				189	7.4	-0.029
Vicia tetrasperma	95	8.4	0.040	342	2.7	0.113						
Viola canina	67	12.8	0.003	47	3.9	0.014	128	26.2	0.016	23	28.2	0.028
Viola hirta	165	5.8	-0.014	52	1.1	-0.014						
Viola lactea	225	87.2	-0.007									
Viola lutea				6	3.1	-0.012	88	3.5	-0.007	36	8.9	-0.016
Viola odorata	78	11.0	0.129	337	1.6	0.053	260	16.4	-0.087	58	5.6	-0.008
Viola palustris	237	81.7	-0.037	142	12.7	-0.111	33	5.5	-0.037	225	2.6	-0.019
Viola reichenbachiana	170	5.3	0.058	301	23.0	-0.032	146	1.8	0.019			
Viola riviniana	208	3.1	-0.004	330	14.8	-0.086	338	2.9	-0.013	325	0.2	-0.019
Viola tricolor	66	11.8	-0.010	277	10.2	0.000				29	16.8	-0.024
Viscum album	253	6.4	0.062									
Vulpia bromoides	160	3.6	0.057	337	5.0	0.045	6	20.9	0.071	84	1.3	0.017
Vulpia ciliata	136	37.5	0.003									
Wahlenbergia hederacea	249	69.4	-0.002				243	10.0	0.002			
Wolffia arrhiza	225	82.2	-0.001									
Zannichellia palustris	245	22.7	-0.017	82	1.3	0.017	237	16.1	0.015	93	16.9	-0.002
